# Transcriptome and coexpression network analysis reveals properties and candidate genes associated with grape (*Vitis vinifera* L.) heat tolerance

**DOI:** 10.3389/fpls.2023.1270933

**Published:** 2023-10-25

**Authors:** Jiuyun Wu, Fuchun Zhang, Guohong Liu, Riziwangguli Abudureheman, Shijian Bai, Xinyu Wu, Chuan Zhang, Yaning Ma, Xiping Wang, Qian Zha, Haixia Zhong

**Affiliations:** ^1^ Turpan Research Institute of Agricultural Sciences, Xinjiang Academy of Agricultural Sciences, Xinjiang Grape Engineering Technology Research Center, Turpan, China; ^2^ The State Key Laboratory of Genetic Improvement and Germplasm Innovation of Crop Resistance in Arid Desert Regions (Preparation), Key Laboratory of Genome Research and Genetic Improvement of Xinjiang Characteristic Fruits and Vegetables, Institute of Horticultural Crops, Xinjiang Academy of Agricultural Sciences, Urumqi, China; ^3^ Xinjiang Uighur Autonomous Region of Grapes and Melons Research Institution, Turpan, China; ^4^ Colleges of Horticulture, Northwest A&F University, Xianyang, China; ^5^ Research Institute of Forestry and Pomology, Shanghai Academy of Agricultural Science, Shanghai, China

**Keywords:** grape, heat stress, RNA-Seq, WGCNA, candidate genes

## Abstract

Temperature is one of the most important environmental factors affecting grape season growth and geographical distribution. With global warming and the increasing occurrence of extreme high-temperature weather, the impact of high temperatures on grape production has intensified. Therefore, identifying the molecular regulatory networks and key genes involved in grape heat tolerance is crucial for improving the resistance of grapes and promoting sustainable development in grape production. In this study, we observed the phenotypes and cellular structures of four grape varieties, namely, Thompson Seedless (TS), Brilliant Seedless (BS), Jumeigui (JMG), and Shine Muscat (SM), in the naturally high-temperature environment of Turpan. Heat tolerance evaluations were conducted. RNA-seq was performed on 36 samples of the four varieties under three temperature conditions (28°C, 35°C, and 42°C). Through differential expression analysis revealed the fewest differentially expressed genes (DEGs) between the heat-tolerant materials BS and JMG, and the DEGs common to 1890 were identified among the four varieties. The number of differentially expressed genes within the materials was similar, with a total of 3767 common DEGs identified among the four varieties. KEGG enrichment analysis revealed that fatty acid metabolism, starch and sucrose metabolism, plant hormone signal transduction, the MAPK signaling pathway, and plant-pathogen interactions were enriched in both between different temperatures of the same material, and between different materials of the same temperature. We also conducted statistical and expression pattern analyses of differentially expressed transcription factors. Based on Weighted correlation network analysis (WGCNA), four specific modules highly correlated with grape heat tolerance were identified by constructing coexpression networks. By calculating the connectivity of genes within the modules and expression analysis, six candidate genes (*VIT_04s0044g01430*, *VIT_17s0000g09190*, *VIT_01s0011g01350*, *VIT_01s0011g03330*, *VIT_04s0008g05610*, and *VIT_16s0022g00540*) related to heat tolerance were discovered. These findings provide a theoretical foundation for further understanding the molecular mechanisms of grape heat tolerance and offer new gene resources for studying heat tolerance in grapes.

## 1 Introduction

Grapes (*Vitis vinifera* L.), as sessile organisms, inevitably encounter various biotic or abiotic stresses during their growth and development (Ju et al., 2020; Ren et al., 2022). Grapes ripen during the summer, coinciding with periods of high temperatures, which significantly impact grape-growing regions. With global climate change, high temperatures have had a severe impact on grape yield and quality (Gouot et al., 2019a; Gouot et al., 2019b). According to the Intergovernmental Panel on Climate Change’s (IPCC) Sixth Assessment Report in 2021, the global average temperature is projected to continue rising. It is estimated that from 2021 to 2040, the global average temperature will increase by 1.5 to 1.6°C compared to the period of 1850-1900 (IPCC, 2021). By the end of this century, the global average temperature is expected to rise by 1.4 to 4.4°C (https://public.wmo.int/en/media/press-release/global-temperatures-set-reach-new-records-next-five-years). The frequency and extent of extreme heat events will continue to increase. High-temperature stress has become one of the major constraints on the health and sustainable development of the grape industry (Mori et al., 2007). Dealing with high-temperature stress will be an unavoidable challenge for the global grape industry (Sun et al., 2018).

High-temperature stress triggers cellular stress responses through signal transduction pathways (Lian et al., 2021). The most sensitive organ in cells affected by high temperatures is the cell membrane (Sadura and Janeczko, 2022). High temperatures can alter the fluidity and structural integrity of the cell phospholipid membrane, leading to protein denaturation and inducing oxidative reactions, stress gene expression, and protein response (Sadura and Janeczko, 2022; Kim et al., 2022). These processes contribute to the manifestation of heat-related phenotypes in plants. However, to adapt to high-temperature environments, plants have evolved ecological habits that enable them to respond and adapt to heat stress promptly. Field observations have shown that sustained high temperatures during the summer can cause grape leaves to curl, lose water, and experience severe sunburn (Mori et al., 2007; Sun et al., 2018). This damage often has a significant impact on normal grape growth and fruit quality (Mori et al., 2007; Sun et al., 2018). High temperatures can also restrict photosynthesis in grape plants, reducing nutrient synthesis and transportation and thereby affecting fruit formation and development (Correia et al., 2021), which can result in smaller and fewer fruits, leading to reduced yield. Additionally, high temperatures can increase the acidity of grape berries, resulting in poor taste and flavor (Mori et al., 2007; Sun et al., 2018). Furthermore, high temperatures may decrease the pigment content in fruits, leading to less vibrant colors (Sun et al., 2018).

In recent years, the widespread application of high-throughput technologies such as RNA-seq-based coexpression network analysis has provided powerful tools and methods for uncovering the molecular characteristics and candidate genes related to plant heat tolerance (Cao et al., 2021; Li et al., 2022b; Meng et al., 2022). Coexpression network analysis is a systems biology approach that constructs gene coexpression networks by analyzing the correlation of gene expression, thereby identifying functionally related gene modules (Langfelder and Horvath, 2008; Tian et al., 2020). In the study of plant heat tolerance, coexpression network analysis can help identify gene sets closely associated with heat tolerance and predict the interaction relationships and regulatory networks of these genes in physiological processes (Liu et al., 2019; Wang et al., 2022). By analyzing key genes in coexpression networks, we can uncover the molecular mechanisms of plants during high-temperature stress, providing potential candidate genes for breeding heat-tolerant varieties (Liu et al., 2019; Wang et al., 2022).

Because grapes are important fruits and wine-making materials, there are significant implications for improving grape yield and quality through the study of heat tolerance (Mori et al., 2007; Sun et al., 2018). Although some grape species have a certain degree of heat tolerance, there is significant variation in heat tolerance among different varieties, and the mechanisms underlying heat tolerance are not well understood. Moreover, prolonged extreme heat stress may permanently affect grape physiological metabolism and yield attributes. Therefore, we selected four grape varieties (detailed information on the materials is provided in **Table S1**) with different levels of heat tolerance for our study and conducted leaf phenotypic and cellular structure analyses as well as RNA-seq sequencing under three temperature conditions during the summer in Turpan, Xinjiang, China. Through clustering analysis, differential expression analysis, GO and KEGG enrichment analysis, expression analysis of transcription factors (TFs), construction of weighted gene coexpression networks, and qRT-PCR, we identified candidate genes for grape heat tolerance. This study provides a theoretical foundation for further understanding the molecular mechanisms of grape heat tolerance and offers new genetic resources for studying heat tolerance in grapes.

## 2 Materials and methods

### 2.1 Plant materials and growth conditions

**Figure 1:**
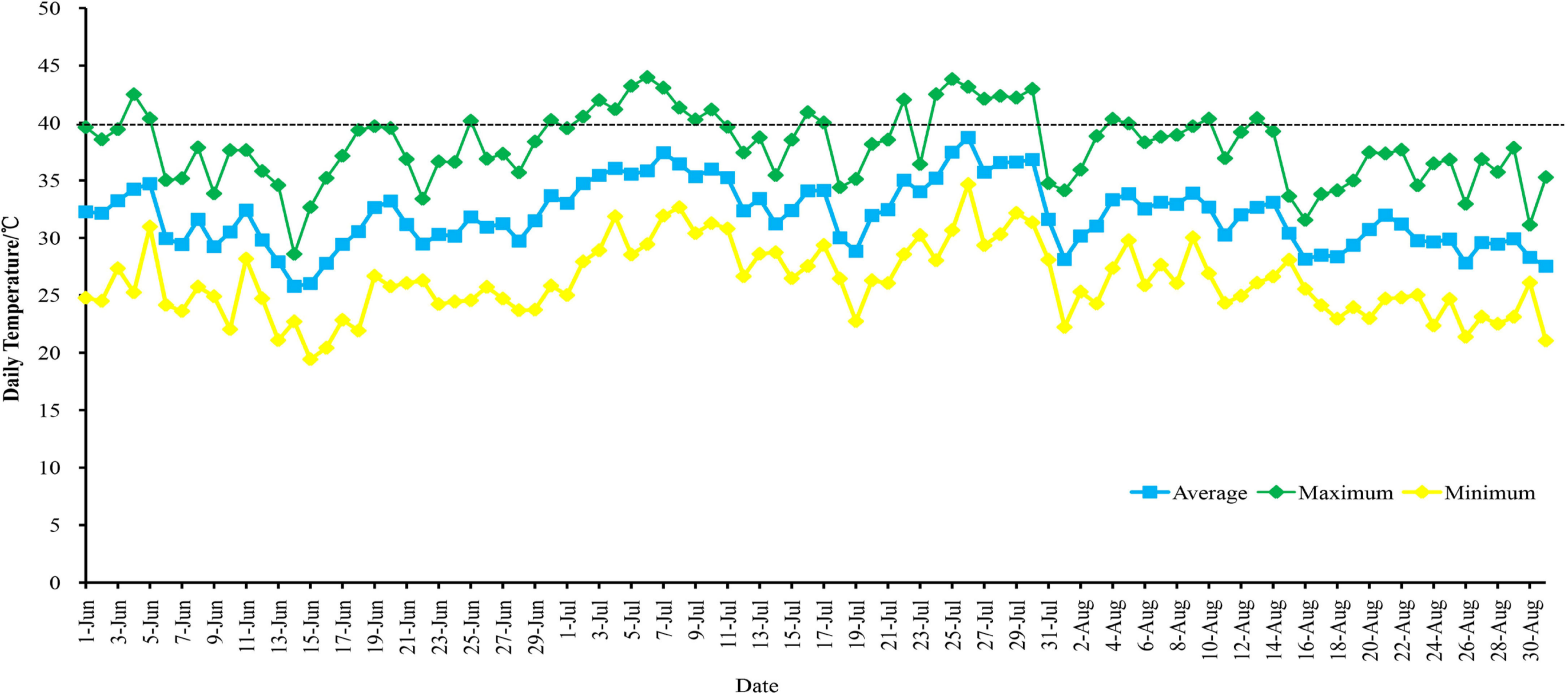
Temperature changes in Turpan from June to August 2021.

In this study, four grape varieties were used. They were planted in the Grape Germplasm Repository of Turpan Agricultural Research Institute, Xinjiang Academy of Agricultural Sciences (89°18′E, 42°53′N). The region has an average annual temperature of 17.6°C, annual precipitation of 12.5 mm, annual sunshine duration of 3109.2 hours, and a frost-free period of approximately 210 days. The grapevines were spaced 1.2 meters within rows and 2.5 meters between rows, arranged in a north-south direction, and were 4 years old. The cultivation employed a ‘V’-shaped trellis system. All grapes received similar irrigation, soil management, pruning, and disease control methods. During the high-temperature period in Turpan in 2021 (June to August), there were 76 days with temperatures above 35°C, including 26 days with temperatures above 40°C (**Figure 1**; **Table S2**). On August 10, 2021, leaf samples were collected at air temperatures of 28°C (T1), 35°C (T2), and 42°C (T3). Leaf samples (the 5th to 9th fully expanded leaves from the top of the canopy, sampling is performed when the temperature is maintained at this temperature for 30 minutes) were collected from each material (four replicates per sample, three for RNA-seq and one for qRT-PCR), rapidly frozen in liquid nitrogen, and stored for subsequent experiments.

### 2.2 Transmission electron microscopy sample preparation

Grape leaf samples (the 5th to 9th fully expanded leaves from the top of the canopy) were collected, excluding the main veins, and cut into small pieces of approximately 1 mm². The leaf samples were then fixed overnight at 4°C in a 2.5% glutaraldehyde solution. Subsequently, the samples were sliced into sections of 70-90 nm using a LEICA EM UC7 ultramicrotome. The sections were stained with lead citrate solution and uranyl acetate-50% ethanol saturated solution for 5-10 minutes each. Finally, the sections were observed under a Hitachi H-7650 transmission electron microscope.

### 2.3 RNA extraction, cDNA library preparation, and sequencing

RNA was extracted using the TRIzol method, and the integrity of the RNA was assessed using 1% agarose gel electrophoresis (Fleige and Pfaffl, 2006). The extracted total RNA was stored at -80°C and transported on dry ice to the PARSUNO Company (Shanghai, China) for sequencing. Fragmentation of the extracted RNA was performed using a PCR plate with a magnetic plate holder (Landolt et al., 2016). The fragmented mRNA was reverse transcribed into cDNA using SuperScript II and random primers (Invitrogen, Carlsbad, California, USA) (Kusser et al., 2006). The obtained data were filtered and quality controlled using fastp software, and the clean data were used for subsequent analysis (Chen et al., 2018). The reads were aligned to the grape genome (http://plants.ensembl.org/Vitis_vinifera/Info/Index) using HISAT2, and StringTie was used for read quantification (Pertea et al., 2016; Kim et al., 2019).

### 2.4 Identification of differentially expressed genes

FPKM (fragments per kilobase of exon per million fragments mapped) was used to measure gene expression levels, which represents the number of reads mapped to exons per kilobase of exon length per million mapped reads. DESeq2 was used to calculate the fold change in gene expression between different samples based on gene expression levels (Liu et al., 2021). DEGs were selected based on the criteria of FDR ≤ 0.01 and absolute log2-fold change ≥ 1. The amino acid sequences of all DEGs were submitted to the KEGG database (https://www.kegg.jp/ghostkoala/) for identification of genes involved in hormone biosynthesis and signal transduction (Kanehisa and Goto, 2000). The grape whole-genome sequence was submitted to the PlantTFDB (http://planttfdb.cbi.pku.edu.cn/) for transcription factor prediction (Jin et al., 2017).

### 2.5 Construction of coexpression networks

The gene expression profiles of the DEGs were subjected to coexpression analysis using the dynamic branch cutting method in the R package WGCNA (Langfelder and Horvath, 2008). To ensure the scale-free distribution of the network, the weight coefficient β was set to 8, which should have a high correlation coefficient close to 0.8 and a certain degree of gene connectivity. The Blockwise Modules function was used to construct the network, resulting in multiple effective modules with varying numbers of genes. Modules with a minimum module size of 30 and a merge cut height of 0.25 were merged if their similarity exceeded 0.75. The module eigengene (ME) vectors and the correlation coefficients between hormone content and different treatment time points were calculated. Specific modules were selected based on a threshold of r > 0.80 and P < 0.05. The genes in the specific modules and predicted transcription factors were used to construct the coexpression network, which was visualized using Cytoscape software (version 3.10) (Shannon et al., 2003).

### 2.6 Quantitative real-time PCR

Total RNA was extracted using the E.Z.N.A. Plant RNA Kit (Omega Bio-Tek, Doraville, GA, USA). The concentration of each RNA sample was measured using a NanoDrop 2000 spectrophotometer (Thermo Fisher Scientific, Waltham, MA, USA). Then, 1 μg of isolated RNA was reverse transcribed into first-strand cDNA using the PrimeScript™ RT Reagent Kit with gDNA Eraser (Takara Bio Inc., Shiga, Japan). qRT-PCR analysis was performed using a Roche LC480 instrument (Roche Diagnostics GmbH, Mannheim, Germany) and SYBR Green (Takara Bio Inc.). Initially, a two-step PCR amplification program was used, with an initial denaturation at 95°C for 30 seconds, followed by 40 cycles of denaturation at 95°C for 5 seconds and annealing at 60°C for 34 seconds. Amplification, melting, and standard curves were generated using Roche LC480 software. geNorm software (https://genorm.cmgg.be/) was used to calculate the relative expression levels of target genes, with *VvGAPDH* as the reference gene. Each program was performed with three biological replicates. The primers used in this study are listed in **Table S3**.

## 3 Results

### 3.1 Effects of high temperature on grape leaf phenotype and cell structure

**Figure 2:**
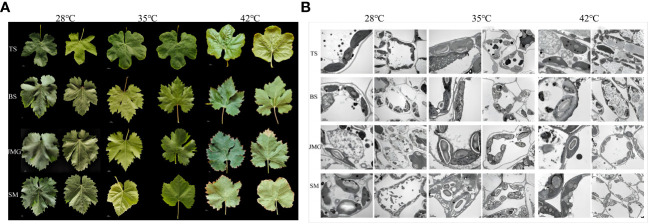
**(A)**, Leaf morphology of four grape varieties at three temperatures. **(B)**, Leaf cell structure of 4 grape varieties at 3 temperatures.

Leaf characteristics directly reflect the degree of high-temperature damage and the ability to resist high-temperature stress (Gong et al., 2023). High temperature stress can cause changes in the morphological characteristics of grape leaves, but different grape varieties exhibit inconsistent phenotypic responses to high-temperature conditions. When the temperature was 28°C, all leaf samples showed no obvious chlorosis (**Figure 2A**). At an air humidity of 35°C, TS exhibited leaf edge curling, SM showed leaf yellowing and chlorosis, and BS and JMG maintained normal leaf morphology without any evident heat damage symptoms (**Figure 2A**). At 42°C, BS and JMG showed marginal desiccation without yellowing, while TS and SM exhibited severe leaf edge curling, desiccation, and pronounced yellowing (**Figure 2A**). High-temperature stress has adverse effects on plant cell structure (Zhao et al., 2019). At 28°C, the cell structures, including the nucleus, vacuole, mitochondria, and chloroplasts, appeared normal in the grape leaf cells of all four materials. The chloroplast membranes and thylakoid membranes were clearly visible with intact structures, the vacuolar membranes had clear edges, and the grana and thylakoid stacks were dense and well-arranged (**Figure 2B**). At 35°C, the thylakoid arrangement was relatively orderly in JMG and TS, while the chloroplasts in BS and SM showed thylakoid swelling or disorganized arrangement with abundant osmiophilic particles (**Figure 2B**). At 42°C, the ultrastructure of leaf cells in all four varieties was damaged to varying degrees, but the chloroplast structure in JMG and BS remained stable with lower levels of damage. TS and SM exhibited an increase in plastoglobules within chloroplasts, and multiple organelle membranes showed fuzzy and damaged appearances (**Figure 2B**). Based on these results, we found that BS and JMG exhibited higher heat tolerance than TS and SM. To further determine the potential molecular mechanisms and candidate genes involved, RNA-seq analysis was conducted.

### 3.2 RNA-seq analysis

**Figure 3:**
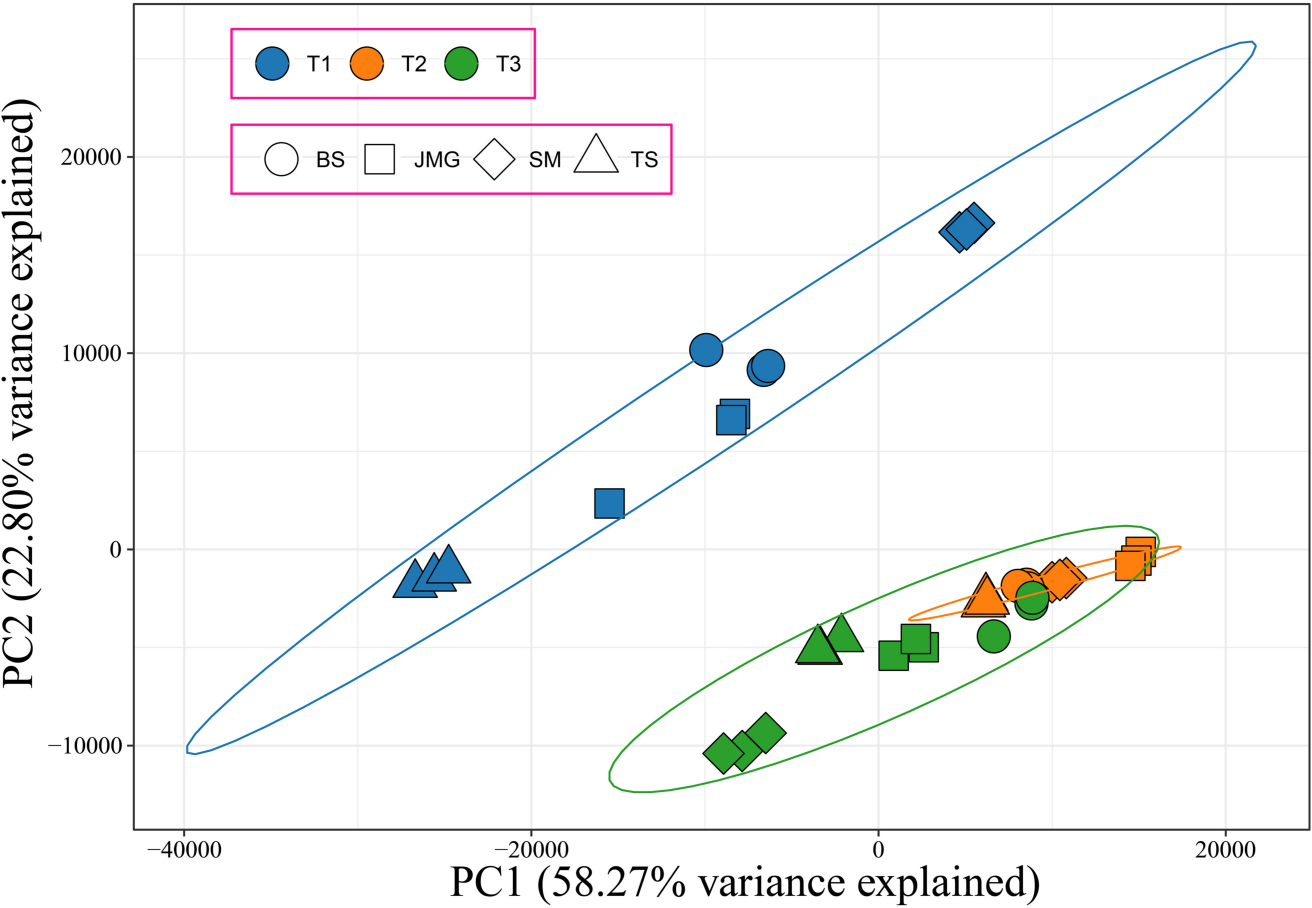
PCA of 36 RNA-seq samples, different colors represent different periods, and different shapes represent different materials.

In total, 36 samples of RNA-seq data were obtained from 4 materials under 3 temperature conditions, resulting in 249.78 Gb of raw data. After filtering, a total of 228.05 Gb clean data were obtained. The effective data obtained from each sample were at least 5.35 Gb, with Q30 base percentages ranging from 91.15% to 93.92% and an average of 93.27%. The alignment rate with the reference genome ranged from 87.70% to 92.64%, with an average alignment rate of 91.31% (**Table S4**). Correlation analysis was initially performed among the samples, and it was found that the correlation among the three replicates of each material at different temperatures exceeded 0.96, indicating a high correlation between the replicates (**Figure S1**). PCA revealed that each replicate clustered together, and the differences between treatments were greater than the differences between materials (**Figure 3**). In summary, the high correlation among the replicates indicates their consistency across different time periods. To confirm the accuracy of the transcriptome expression profile, six randomly selected genes were subjected to qRT-PCR analysis with three independent replicates, and the results showed similar expression patterns to those observed in RNA-seq, confirming the reliability of the RNA-seq data for further analysis.

### 3.3 Differential expression analysis

**Figure 4:**
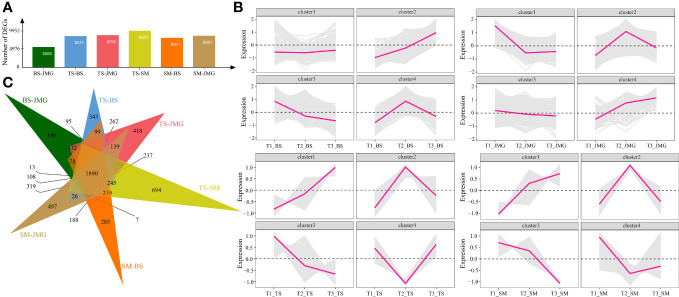
**(A)** Number of DEGs between different materials. **(B)** Line chart of DEG expression patterns between different materials. **(C)** DEG Wayne diagrams between different materials.

First, differential expression analysis was performed among different materials under the same temperature. There were 5508 DEGs between BS and JMG, 8511 DEGs between TS and BS, 8756 DEGs between TS and JMG, 9952 DEGs between TS and SM, 8021 DEGs between SM and BS, and 8601 DEGs between SM and JMG (**Figure 4A**). Using the k-means clustering method, a total of 4 statistically significant clusters were identified among the 1890 commonly differentially expressed genes (**Figures 4B, C**). In BS, although Cluster 1 showed a trend of downregulation followed by upregulation, the overall expression trend change was not significant. Cluster 2 exhibited an increasing expression level with increasing temperature, while Cluster 3 showed the opposite expression trend compared to Cluster 2. Cluster 4 displayed an initial increase followed by a decrease in expression (**Figure 4B**). In JMG, Cluster 1 showed a trend of downregulation followed by a plateau, Cluster 2 exhibited an expression trend of initial increase followed by a decrease, Cluster 3 showed a gradual decrease in expression with increasing temperature, and Cluster 4 displayed increasing expression with increasing temperature (**Figure 4B**). In TS, Cluster 1 exhibited increasing expression with increasing temperature, Cluster 2 showed a trend of upregulation followed by downregulation, Cluster 3 displayed a downregulation trend, and Cluster 4 showed the opposite trend compared to Cluster 2 (**Figure 4B**). In SM, Cluster 1 exhibited increasing expression with increasing temperature, Cluster 2 showed a trend of upregulation followed by downregulation, Cluster 3 displayed a downregulation trend, and Cluster 4 showed a slight increase after an initial decrease (**Figure 4B**).

**Figure 5:**
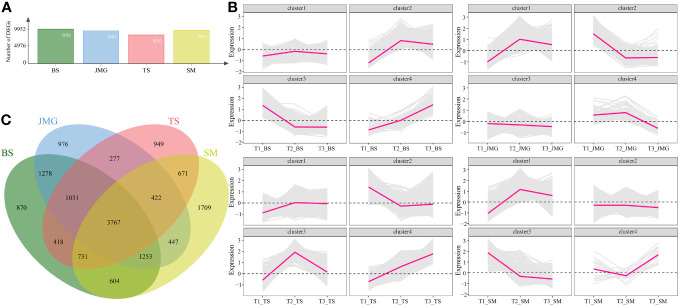
**(A)** Number of DEGs between different temperatures. **(B)** Line chart of DEG expression patterns between different temperatures. **(C)** DEG Wayne diagrams between different temperatures.

We also performed differential expression analysis on the same material under different temperatures. There were 9952 DEGs in BS, 9451 DEGs in JMG, 8266 DEGs in TS, and 9604 DEGs in SM. The DEGs between heat-tolerant and heat-sensitive materials did not differ significantly (**Figure 5A**). Using the k-means clustering method, a total of 4 statistically significant clusters were identified among the 3767 common DEGs (**Figures 5B, C**). In BS, although Cluster 1 showed a trend of upregulation followed by downregulation, the overall expression trend change was not significant. Cluster 2 exhibited an expression trend of an initial increase followed by a decrease, while Cluster 3 showed decreasing expression with increasing temperature, and Cluster 4 displayed increasing expression with increasing temperature (**Figure 5B**). In JMG, Cluster 1 showed an initial increase followed by a decrease in the expression trend, Cluster 2 exhibited a gradual decrease after an initial decrease, Cluster 3 showed a slight decrease with temperature change, and Cluster 4 displayed a trend of initial stability followed by a decrease (**Figure 5B**). In TS, Cluster 1 showed an initial increase followed by relatively stable expression, Cluster 2 exhibited a trend of downregulation followed by a plateau, Cluster 3 showed an initial increase followed by a decrease in the expression trend, and Cluster 4 exhibited increasing expression with increasing temperature (**Figure 5B**). In SM, Cluster 1 showed an initial increase followed by a slight decrease in expression, Cluster 2 exhibited a slight decrease in expression, Cluster 3 displayed a downregulation trend, and Cluster 4 showed a slight decrease followed by an increase in expression (**Figure 5B**).

### 3.4 GO and KEGG enrichment analysis of DEGs

**Figure 6:**
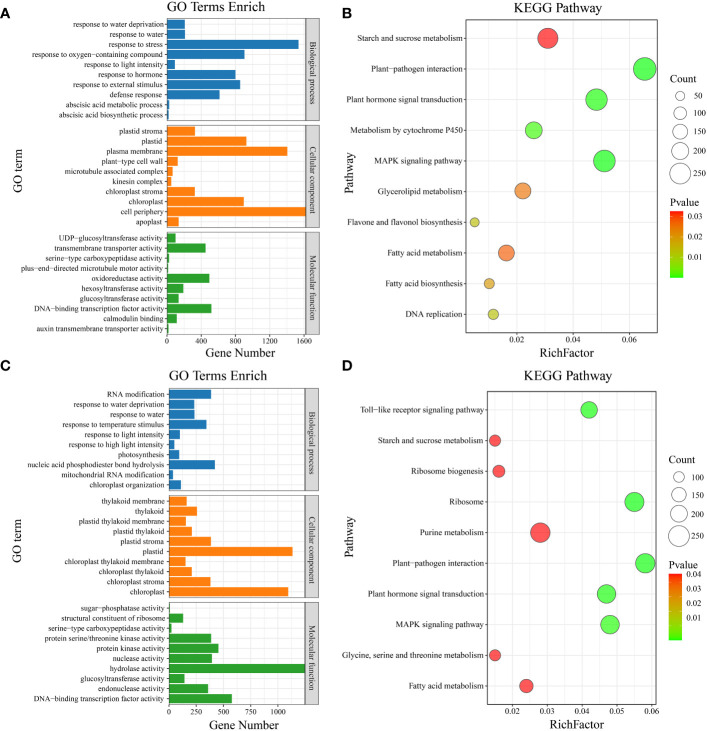
**(A)** Material-to-material DEG GO enrichment analysis. **(B)** Material-to-material DEG KEGG enrichment analysis. **(C)** GO enrichment analysis of DEGs at different temperatures. **(D)** KEGG enrichment analysis of DEGs at different temperatures.

Gene Ontology (GO) enrichment analysis was performed on the common DEGs between materials (**Figure 4**). The significantly enriched GO terms included defense response, response to oxygen-containing compound, response to light intensity, abscisic acid metabolic process, response to external stimulus, response to stress, response to hormone, response to water, response to water deprivation, and abscisic acid biosynthetic process (**Figure 6A**). KEGG pathway enrichment analysis of the common DEGs between materials revealed enrichment in plant-pathogen interaction, MAPK signaling pathway, plant hormone signal transduction, metabolism by cytochrome P450, DNA replication, flavone and flavonol biosynthesis, fatty acid biosynthesis, glycerolipid metabolism, fatty acid metabolism, and starch and sucrose metabolism (**Figure 6B**). GO enrichment analysis was also conducted on the material-specific DEGs (**Figure 5**). The significantly enriched GO terms within each material included RNA modification, nucleic acid phosphodiester bond hydrolysis, mitochondrial RNA modification, response to light intensity, response to temperature stimulus, photosynthesis, response to water, response to water deprivation, chloroplast organization, and response to high light intensity (**Figure 6C**). KEGG pathway enrichment analysis of the material-specific DEGs showed enrichment in ribosome, plant-pathogen interaction, Toll-like receptor signaling pathway, plant hormone signal transduction, MAPK signaling pathway, ribosome biogenesis, purine metabolism, glycine, serine and threonine metabolism, fatty acid metabolism, and starch and sucrose metabolism (**Figure 6D**).

### 3.5 TF expression analysis

**Figure 7:**
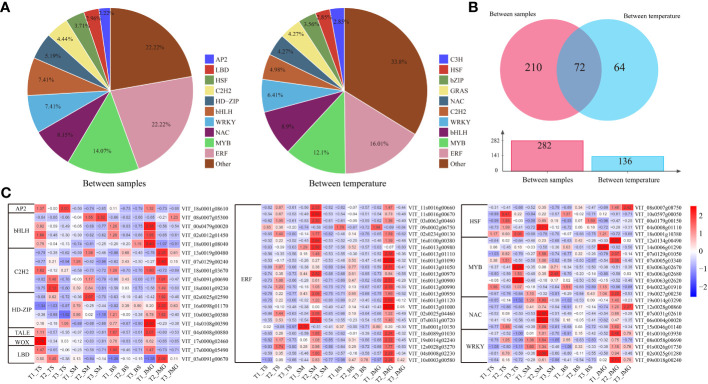
**(A)** TF pie chart of material and temperature differences. **(B)** TF Wayne diagram for differences between materials and different temperatures. **(C)** Heatmap of differential TF expression.

A total of 282 differentially expressed transcription factors (TFs) were identified between materials, and 136 differentially expressed TFs were identified under different temperature conditions, mainly including ERF, MYB, NAC, bHLH, and WRKY (**Figures 7A, B**). The expression patterns of differentially expressed TF genes were visualized using a heatmap, and most TFs showed the highest expression under T2 temperature conditions (**Figure 7C**). The expression levels of bHLH, C2H2, and HD-ZIP were predominantly higher in heat-tolerant materials, while ERF exhibited the highest expression at the T2 temperature in SM and JMG (**Figure 7C**). HSF showed high expression in both heat-tolerant materials and heat-sensitive materials, indicating the complex heat tolerance mechanism of HSF in grapes (**Figure 7C**). The expression patterns of MYB, NAC, and WRKY were also complex, similar to HSF, suggesting that more experiments are needed to validate and elucidate the roles and functions of these TFs in the heat tolerance process of grapes.

### 3.6 WGCNA exploration of heat-tolerant hub genes

**Figure 8:**
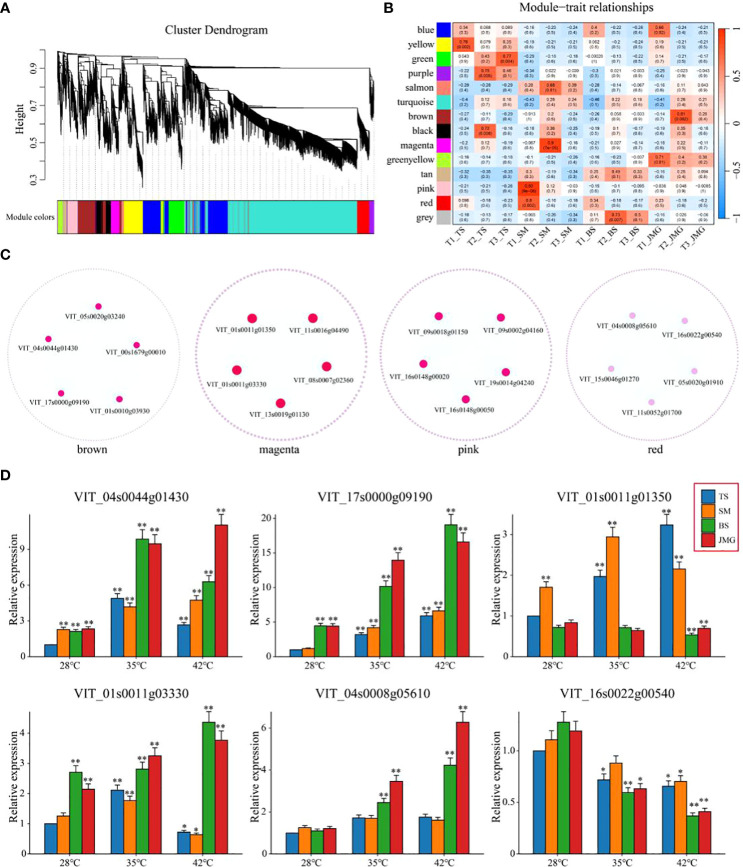
**(A)** Hierarchical clustering tree of genes based on coexpression network analysis. **(B)** Heatmap of correlation and significance between modules and materials at different temperatures. **(C)** Gene coexpression networks within specific modules. **(D)** qRT-PCR of grape heat-resistant hub genes. The results are presented as the means ± SDs (n = 3, *P < 0.05,**P < 0.01).

Weighted gene coexpression network analysis (WGCNA) was performed on a total of 5,156 commonly differentially expressed genes (DEGs) between materials and within materials to construct a coexpression network associated with heat tolerance in grapes (β soft thresholding parameter set to 8, scale-free R^2 > 0.80), resulting in 14 expression modules (**Figure 8A**). Based on the correlation results between modules and materials, the brown module showed a significant correlation with JMG at T2 temperature, the magenta module showed a significant correlation with SM at T2 temperature, and the pink and red modules showed a significant correlation with SM at T1 temperature (**Figure 8B**). The brown, magenta, pink, and red modules were selected to construct a gene interaction network and identify hub genes. Cytoscape was used for network visualization (**Figure 8C**). Five hub genes were determined for each module, resulting in a total of 20 hub genes (**Figure 8C**). The expression patterns of 20 hub genes at different temperatures were detected using qRT-PCR (**Figure 8D**; **Figure S1**). Among them, only *VIT_04s0044g01430*, *VIT_17s0000g09190*, *VIT_01s0011g01350*, *VIT_01s0011g03330*, *VIT_04s0008g05610* and *VIT_16s0022g00540* showed differential expression between heat-tolerant and heat-sensitive materials (**Figure 8D**). Among these, four genes (*VIT_04s0044g01430*, *VIT_17s0000g09190*, *VIT_01s0011g03330* and *VIT_04s0008g05610*) exhibited higher expression in heat-tolerant materials, suggesting their potential role as positive regulatory genes in heat tolerance, while two genes (*VIT_01s0011g01350* and *VIT_16s0022g00540*) showed higher expression in heat-sensitive materials, indicating their potential role as negative regulatory genes in heat tolerance (**Figure 8D**). In conclusion, we identified six candidate genes related to heat tolerance through qRT-PCR. These findings provide a theoretical basis for a deeper understanding of the molecular mechanisms underlying heat tolerance in grapes and offer new genetic resources for heat tolerance research in grapes.

## 4 Discussion

The impact of high-temperature stress on plant leaves directly reflects the plant’s ability to cope with and resist heat stress (Zoong Lwe et al., 2021). Different varieties exhibit varying responses to high temperatures, with some maintaining better morphology and cellular structure under high-temperature conditions, demonstrating higher heat tolerance, while others are more susceptible to the effects of heat stress (Panigrahi et al., 1996; Li et al., 2022b). This variation may be attributed to genetic differences between varieties and variations in the plant’s own adaptability to heat stress (Sadura and Janeczko, 2022; Kim et al., 2022). Leaf morphological changes and cellular structural damage induced by high-temperature stress may be associated with the interactions of multiple physiological and biochemical mechanisms (Hasanuzzaman et al., 2013; Sadok et al., 2021). For instance, leaf curling and yellowing may result from water imbalance within leaf cells and restricted photosynthesis (Kim et al., 2022; Li et al., 2022a). The reduced transpiration caused by high temperatures leads to decreased water evaporation and accumulation of moisture within the leaves, potentially causing leaf curling (Hasanuzzaman et al., 2013; Sadok et al., 2021). Simultaneously, the inhibition of photosynthesis by high temperatures can lower the chlorophyll content in leaves, resulting in leaf yellowing (Panigrahi et al., 1996; Li et al., 2022a). On the other hand, high-temperature stress may cause lipid peroxidation of cell membranes and ion imbalance, leading to cellular structural and functional damage (Hasanuzzaman et al., 2013; Sadok et al., 2021). Lipid peroxidation of the cell membrane disrupts its integrity, resulting in substance leakage and ion imbalance, which affects normal cell metabolism and function (Crockett, 2008; Thuwanut et al., 2009). Furthermore, high-temperature stress may also increase intracellular oxidative stress, further damaging cellular structure (Crockett, 2008; Thuwanut et al., 2009). Under different high-temperature stress conditions, we observed changes in the phenotypic characteristics of grape leaves and found that different grape varieties exhibited distinct morphological changes under high-temperature environments (**Figure 2**). This finding indicates variations in sensitivity and resistance to high temperatures among different varieties. Heat-tolerant materials may be able to regulate water balance and photosynthesis under high-temperature conditions, thereby reducing leaf dehydration and yellowing. Overall, the observed changes in the phenotypic characteristics of grape leaves and cellular structural damage under high-temperature stress reflect the plant’s response and resistance level to heat stress.

Research on heat tolerance-related pathways in plants has always been a topic of intense interest in the fields of plant biology and agricultural science. With increasing attention being paid to global climate warming and heat stress, significant progress has been made in understanding the mechanisms of plant heat tolerance (Gouot et al., 2019a; Gouot et al., 2019b). Heat shock proteins (HSPs) are a class of proteins that are induced under high-temperature stress and help cells cope with protein instability and aggregation caused by heat (Li and Howell, 2021). The heat shock protein pathway includes processes such as synthesis, folding, and localization of HSPs, which protect cells from damage by maintaining protein stability and function under high-temperature stress (Khan et al., 2020). Moisture is one of the key factors in plant resistance to heat stress. Plants maintain water balance by regulating root water uptake and transpiration (Egawa et al., 2020). Studies have shown that some heat-tolerant plants have stronger root water uptake capacity and water retention ability, which can reduce water evaporation and dehydration under high-temperature environments, thereby lowering leaf temperature and damage. Plant hormones play an important role in regulating plant responses to high-temperature stress (Egawa et al., 2020). For example, hormones such as gibberellins, abscisic acid, and ethylene are involved in regulating plant growth, development, and stress tolerance. Studies have shown that the synthesis, signal transduction, and regulatory mechanisms of these hormones under high-temperature stress have a significant impact on plant heat tolerance (Castroverde and Dina, 2021). Through enrichment analysis, we also identified the important roles of signaling pathways such as fatty acid metabolism, starch and sucrose metabolism, plant hormone signal transduction, the MAPK signaling pathway, and plant-pathogen interactions in grape heat tolerance processes.

In recent years, numerous heat-tolerant TFs have been identified in plants, and their regulation of target genes plays a crucial role in enhancing plant heat tolerance (Sahebi et al., 2018; Chauve et al., 2021). Heat shock transcription factors (HSFs) are considered to play a decisive role in this process. Studies in Arabidopsis have found that HSFA3, in addition to *HSFA2*, is another member of the HSF family that functions in heat stress memory (Friedrich et al., 2021). *HSFA3* can directly activate or maintain the hypermethylation of histone H3K4 to regulate the expression of genes related to heat stress memory (Friedrich et al., 2021). WRKY transcription factors play critical roles in plant responses to biotic and abiotic stresses. While there are reports demonstrating that overexpression of *OsWRKY11* under the promoter of the heat shock protein gene *HSP101* can enhance heat tolerance in rice, the *WRKY10* transcription factor negatively regulates rice heat tolerance through the regulation of ROS balance and hypersensitive responses, with its interacting protein *VQ8* playing an antagonistic role (Wu et al., 2009). In our study, we found that ERF, NAC, WRKY, MYB, and bHLH transcription factors in grapes may be associated with heat tolerance (**Figure 7**), providing reliable candidate genes for further investigation of the molecular mechanisms underlying heat tolerance in grapes.

Coexpression network analysis is a systems biology approach that involves analyzing the correlation of gene expression and constructing gene coexpression networks to discover functionally related gene modules. This analysis helps us identify candidate genes closely associated with our research (Langfelder and Horvath, 2008). Using WGCNA, 15 TFs related to poplar leaf blight, including *ATWRKY75*, *ANAC062*, *ATMYB23* and *ATEBP*, were identified, and these TFs exhibited high connectivity in the network (Wang et al., 2023). In potatoes, WGCNA identified 40 key candidate genes associated with development (Wei et al., 2023). In pepper, a key heat-tolerant gene, *CcBES1*, was discovered through WGCNA. *CcBES1* binds to the HSF promoter region in yeast, thereby regulating heat tolerance (Mumtaz et al., 2023). In our study, using transcriptomic data from leaf samples of grapes at different temperatures, we identified four highly significant gene modules through WGCNA, and a total of 20 heat stress-responsive genes were discovered. qRT-PCR analysis revealed that the expression of *VIT_04s0044g01430*, *VIT_17s0000g09190*, *VIT_01s0011g03330* and *VIT_04s0008g05610* was higher in heat-tolerant materials than in heat-sensitive materials. These four genes are potential positive regulators of heat tolerance in grapes. Among them *VIT_04s0044g01430* encode a Polyadenylate-binding protein (PABP) protein, and the functional annotation shows that it is mainly involved in response to light stimulus. *VIT_17s0000g09190* encodes a Phox and Bem1 (PB1) protein, and functional annotations show that it is primarily involved in the cellular lipid metabolic process. *VIT_01s0011g03330* encodes an Increased sodium tolerance protein 1 (IST1) protein, and the functional annotations show that it is primarily involved in Response to oxidative stress. *VIT_04s0008g05610* encodes a Coiled-Coil Domain-Containing (CCDC) protein, and the functional annotation shows that it is primarily involved in the Starch biosynthetic process. Conversely, the expression of *VIT_01s0011g01350* and *VIT_16s0022g00540* was higher in heat-sensitive materials than in heat-tolerant materials, suggesting that these two genes may be negative regulators of heat tolerance in grapes. *VIT_01s0011g01350* encodes a Valine-glutamine (VQ) protein, a gene with unknown function, *VIT_16s0022g00540* encodes a Glycerol-3-phosphate permease (G3Pp) protein, and functional annotations show that it is primarily involved in Transmembrane transport. Although the functions of these genes in grape heat tolerance require further validation, subsequent in-depth research can be conducted using biotechnological methods to elucidate their molecular mechanisms.

## 5 Conclusion

In summary, this study evaluated heat tolerance in four grape varieties and examined cellular structures in the naturally high-temperature environment of Turpan. RNA-seq analysis was performed, and the results showed that there were minimal differences in DEGs between the BS and JMG heat-tolerant materials, with a total of 1,890 DEGs identified. Additionally, the number of differentially expressed genes within materials was not significantly different, resulting in a total of 3,767 common DEGs. KEGG enrichment analysis revealed the enrichment of pathways such as fatty acid metabolism, starch and sucrose metabolism, plant hormone signal transduction, MAPK signaling pathway, and plant-pathogen interaction, both between and within the materials. By constructing a coexpression network, four specific modules highly associated with grape heat tolerance were identified, and six candidate genes related to heat tolerance were selected through qRT-PCR. These research findings provide a theoretical basis for a deeper understanding of the molecular mechanisms underlying grape heat tolerance and offer new genetic resources for studying grape heat tolerance.

## Data availability statement

The original contributions presented in the study are publicly available. This data can be found here: https://www.ncbi.nlm.nih.gov/sra/PRJNA914878.

## Author contributions

JW: Data curation, Formal Analysis, Writing – original draft, Writing – review & editing. FZ: Methodology, Supervision, Validation, Writing – review & editing. GL: Methodology, Supervision, Validation, Writing – review & editing. RA: Methodology, Supervision, Validation, Writing – review & editing. SB: Methodology, Supervision, Validation, Writing – review & editing. XYW: Methodology, Supervision, Validation, Writing – review & editing. CZ: Methodology, Supervision, Validation, Writing – review & editing. YM: Methodology, Supervision, Validation, Writing – review & editing. XPW: Methodology, Supervision, Validation, Writing – review & editing. QZ: Data curation, Formal Analysis, Methodology, Supervision, Validation, Writing – original draft, Writing – review & editing. HZ: Data curation, Formal Analysis, Methodology, Supervision, Validation, Writing – original draft, Writing – review & editing.

## Supplementary material

Supplementary Figure 1 Correlation analysis of 36 RNA-seq samples.

Supplementary Figure 2 qRT-PCR of grape heat-resistant hub genes. The results are presented as the means ± SDs (n = 3).
