# Supplementary figures and images for: Transcriptome and coexpression network analysis reveals properties and candidate genes associated with grape (Vitis vinifera L.) heat tolerance
Source: Front Plant Sci. 2023 Oct 25;14:1270933. doi: 10.3389/fpls.2023.1270933 (PMC10643163; doi:10.3389/fpls.2023.1270933)

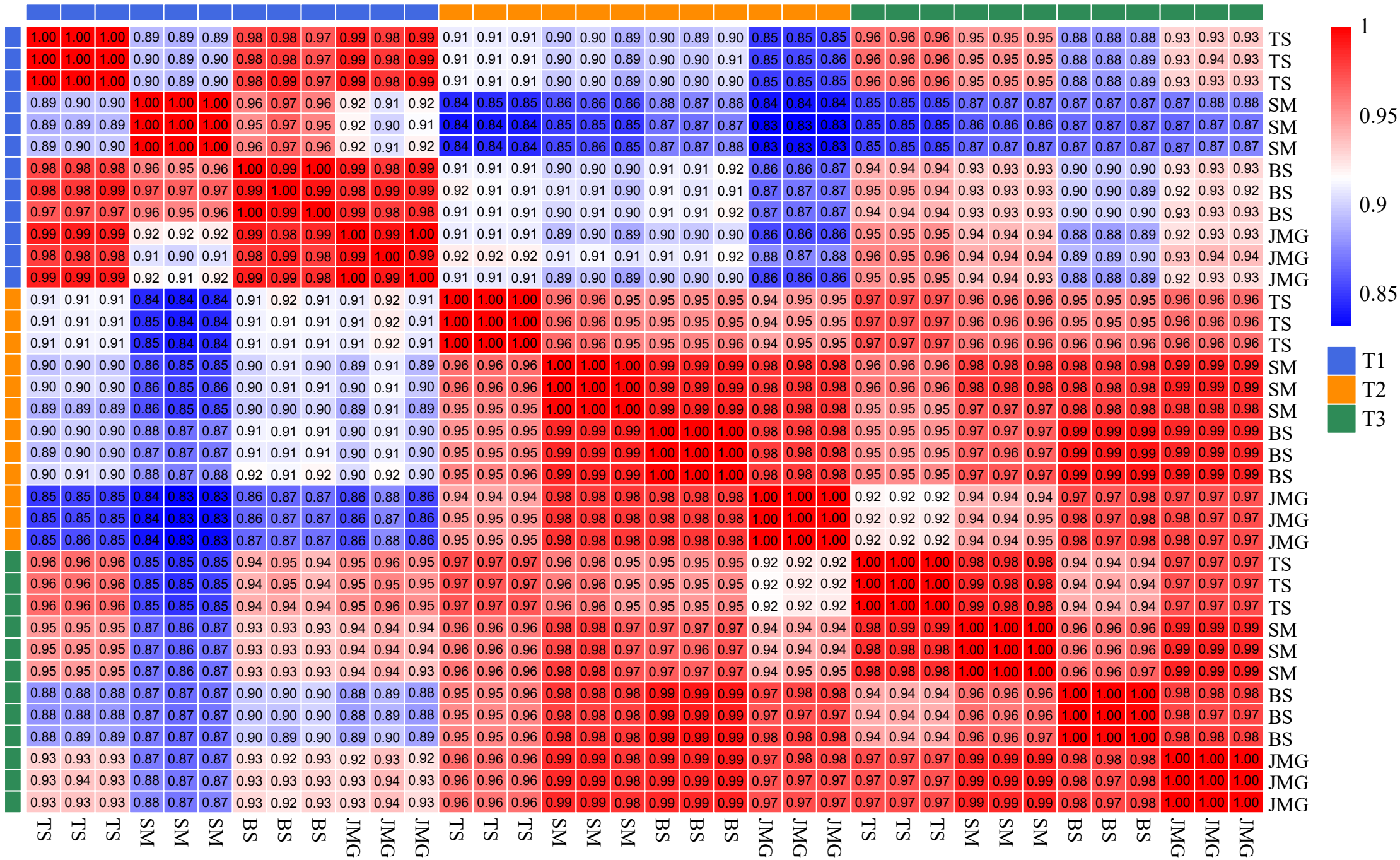

Supplement: Supplementary Figure 1 — Correlation analysis of 36 RNA-seq samples. [file DataSheet_1.pdf]

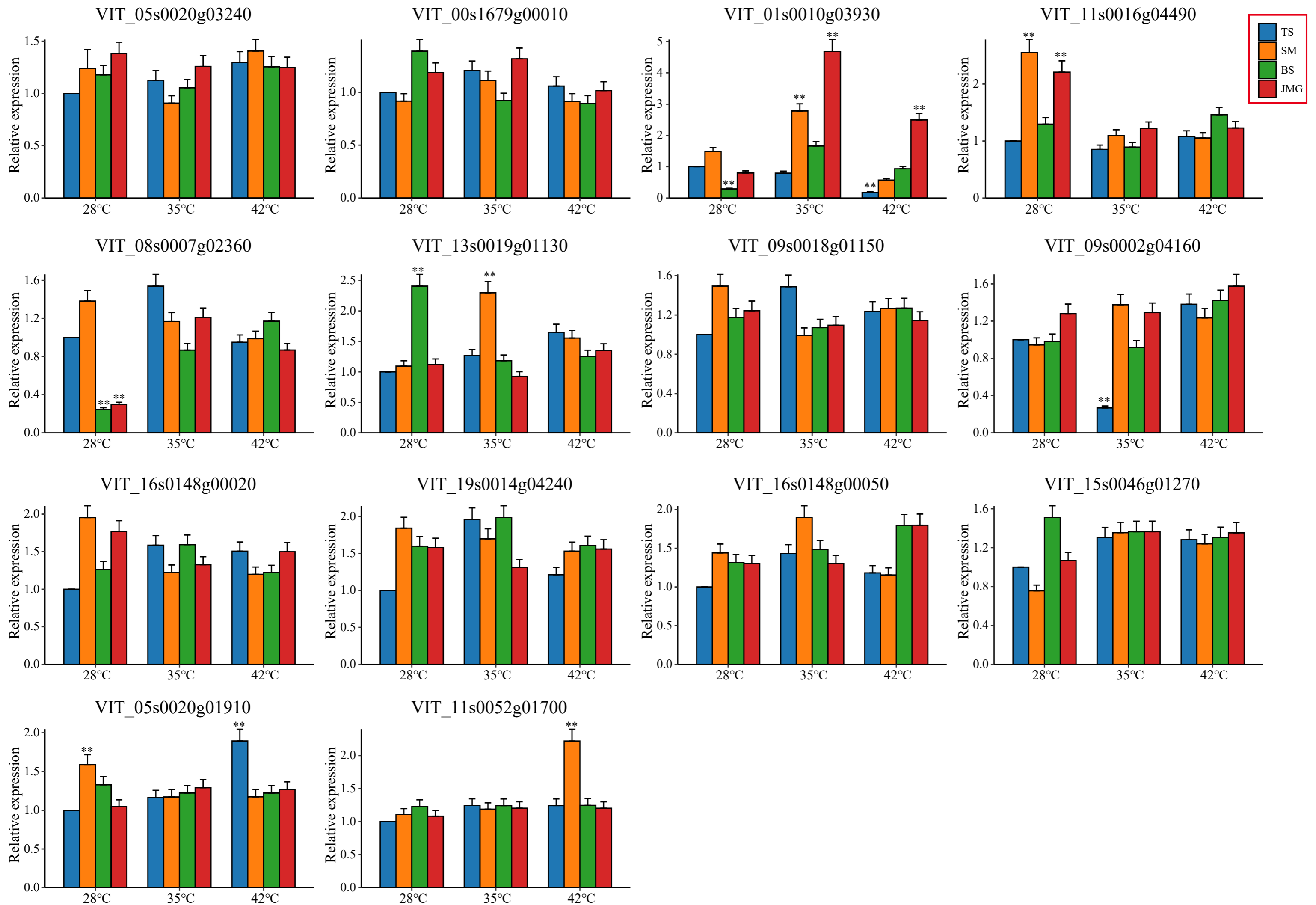

Supplement: Supplementary Figure 2 — qRT-PCR of grape heat-resistant hub genes. The results are presented as the means ± SDs (n = 3). [file DataSheet_2.pdf]
